# Depression and Medierranean diet: analysis of the PREDIDEP randomised trial

**DOI:** 10.1192/j.eurpsy.2024.412

**Published:** 2024-08-27

**Authors:** B. M. Cabrera Suárez, A. Sánchez Villegas, J. L. Hernández Fleta, P. Molero Santos, J. T. R. Sosa

**Affiliations:** ^1^Hopsital Universitario de Gran Canaria Dr. Negrín, Las Palmas de Gran Canaria; ^2^Universidad Pública de Navarra, Navarra, Spain

## Abstract

**Introduction:**

Unipolar depression is a growing global Public Health challenge. During last years, life factors such as diet, have been identified as a target for the development of adjunctive treatment that could reduce the rates of depression. The Mediterranean Diet (MD) is one of the most studied dietary factors that has been inversely associated with depression (Rahe et al. Eur J Nutr. 2014;53:997–1013). The PREDIDEP study is an ongoing secondary prevention trial aimed at assessing the effect of a MD enriched with extra virgin olive oil (EVOO) on depression recurrence (Sánchez-Villegas et al. BMC Psychiatry. 2019 Feb 11;19(1):63).

**Objectives:**

This study aims to assess the effectiveness of a remote Mediterranean diet–based nutritional intervention in the context of a trial of depression.

**Methods:**

The PREDIDEP study is a 2-year multicenter, randomized, single-blinded trial designed to analyse the effect of the MD enriched with extra virgin olive oil (EVOO) on the prevention of depression recurrence. The inervention group received phone contacts with dietist and had access to web-based information, and the control group had usual care for depressed patients. The 14-item MD Adherence Screener (MEDAS) questionnaire and a semiquantitative food frequency questionnaire (FFQ) were collected by dietitians at baseline and at 1-year and 2-year of follow-up. We used mixed effects linear models to assess changes in nutritional variables according to the group of intervention. The trial was registered at ClinicalTrials.gov NCT03081065.

**Results:**

We observed that participants in the MD group increased their adherence to MD (between-group difference: 2.50; 95% CI 1.88-3.12; p<0.001) after one and two years (between-group difference: 2.57; 95% CI 1.93-3.22; p<0.001) of intervention compared with control group.

**Table:** 

MEDAS questionnaire	Control, mean (95% CI)	Intervention, mean (95% CI)	Between group difference, mean (95% CI)	P value
Baseline	6.96 (6.54-7.39)	7 (6.63-7.39)	N/A	N/A
1 year	7.2 (6.82-7.58)	9.74 (9.3-10.18)	N/A	N/A
1-year change	0.23 (-0.19-0.65)	2.74 (2.28-3.19)	2.50 (1.88-3.12)	<0.001
2 years	7.06 (6.66-7.46)	9.68 (9.28-10.07)	N/A	N/A
2-years change	0.10 (-0.38-0.58)	2.67 (2.24-3.1)	2.57 (1.93-3.22)	<0.001

Calculated using mixed-effect models with center as random factor.

P value between group intervention difference.

N/A: not applicable.

MEDAS: Mediterranean Diet Adherence Screener

**Conclusions:**

We found that this multifaceted remote nutritional intervention is a useful tool kit to maintain the quality of the diet according to the goals of the MD among patients at risk of depression.

**Disclosure of Interest:**

None Declared

